# Author's misconduct inviting risk: Duplicate publication

**DOI:** 10.4103/0301-4738.57142

**Published:** 2009

**Authors:** B K Nayak

**Affiliations:** P. D. Hinduja National Hospital and MRC, Veer Savarkar Marg, Mumbai-400 016, India. E-mail: editor@ijo.in

Duplicate publication frequently occurs in biomedical literature and is considered as author's misconduct.[1] A publication is considered duplicate if it significantly overlaps another article in which, at least one of the authors is common to both, whereas it becomes plagiarism if none of the authors are common. Redundant, repetitive, dual, fragmented and disaggregated publications are various terminologies used to describe duplicate publication.[2] Sometimes authors slice one research into two or more papers and make them into many publishable units. This is considered justifiable if done with a valid reason of answering a different question. However, this needs to be properly cross-referenced and should be stated clearly in the ‘materials and methods’ section of the manuscript, as well as in the covering letter. The intentions of authors become suspect when they try to avoid transparency. Various types of duplicate publications have been enumerated by Alphonso *et al*.[3]

Why do duplicate publications occur? Various justifications are given by authors facing the charge of duplicate publication. It has been nicely enumerated by Tobin in his editorial.[2] They include “we did not read the instructions”, “we wanted to reach a different audience”, “our failure to cross-reference the article was a simple oversight”, “we perceive the overlap to be much less than the reviewer or editor thinks” and “we now see that we broke the rules, but this was never our intent”. Tobin also, justifiably, writes that one answer which is probably applicable to most of them is never given, “we thought this would be a good way of lengthening our curriculum vitae”. This was confirmed in a survey wherein 75% of editors and 95% of authors felt that duplicate publications occur due to the pressure on the authors to publish.[4] In the same survey there was consensus amongst the editors and authors that the journal was not doing enough to identify, publicize, criticize and punish cases of duplicate publication.

What are the drawbacks of duplicate publication? Peer-reviewed journals are perceived to provide new and authentic information. Duplicate publication hurts the prestige of the journal as the assurance of giving novel ideas to the readers is not fulfilled. It also violates the copyright of the previous journal, since the authors have already signed agreement of copyright transfer of contents to them and are not in a position to transfer this right again to the new journal, without the previous journal's consent. Duplicate publications are also declared unethical since they utilize the journal's limited resources, waste time of editors and reviewers, increase the work of the indexing system, artificially inflate the author's bio-data, and above all skew the medical evidence which in turn affects the meta- analysis.[5]

Authors can avoid duplicate publication if they follow the two fundamental policies of “awareness” and “openness”.[6] Firstly, they should be aware of issues related to duplicate publication and should judge for themselves in an unbiased manner whether their manuscript falls in the category of duplicate publication or not. Secondly, they should be transparent in providing cross-references to their own published (related) work and submit (related) unpublished data to the new journal. Further, these matters should be left to the discretion of the Editor of the Journal. The difficulty arises when this information is withheld by the authors. Authors should also realize that in the electronic era there are very high chances that duplication will get detected in due course. Authors should also read the author's instructions and copyright transfer form carefully before submitting the paper. If the author is uncomfortable about any aspect he/she should take advice from a knowledgeable and qualified person. Remember, not publishing will do no harm but any punitive action for unethical behavior will do irreparable damage to your prestige. Be reminded of Scott Fitzgerald's maxim: Write because you have something to say, not because you want to say something.[2]

Many journals are concerned about this unethical practice of authors.[7–17] If it is detected during the time of review, the job of the Editor becomes relatively easy. He only has to inform the other journal's Editor about this misconduct and stop further processing of the article. Editors have to abide by the guidelines provided by committee of publication ethics (COPE) if the article is detected to be duplicated after it has been published.[1] The Indian journal of Ophthalmology (IJO) clearly mentions in its copyright transfer form “Neither this manuscript nor one with substantially similar content under my/our authorship has been published or is being considered for publication elsewhere, except as described in the covering letter” which is mandatory for authors to sign and submit, for possible publication in IJO.

The foundation of scientific publication is based on trust, honesty and credibility. Originality is the key factor in scientific progress and if the authors cannot assure this then one develops mistrust towards the contents of the article. The ultimate responsibility for the integrity of the publication rests on the shoulders of the authors.[18] In the larger interest of mankind these aberrations must be dealt with firmly and immediately.
